# Factors Associated with Adverse Cardiovascular Events in Cancer Patients Treated with Bevacizumab

**DOI:** 10.3390/jcm9082664

**Published:** 2020-08-18

**Authors:** Doan T. M. Ngo, Trent Williams, Sophie Horder, Leonard Kritharides, Janette Vardy, Hiren Mandaliya, Ina I. C. Nordman, James Lynam, Tony Bonaventura, Aaron L. Sverdlov

**Affiliations:** 1 School of Biomedical Sciences and Pharmacy, University of Newcastle, Callaghan, NSW 2308, Australia; Sophie.Horder@uon.edu.au; 2 Hunter Medical Research Institute, New Lambton Heights, NSW 2305, Australia; 3 Hunter Cancer Research Alliance, Waratah, NSW 2298, Australia; Hiren.Mandaliya@calvarymater.org.au; 4 Hunter New England Local Health District, Newcastle, NSW 2305, Australia; Trent.Williams@health.nsw.gov.au (T.W.); James.Lynam@calvarymater.org.au (J.L.); 5 Department of Cardiology, Concord Repatriation General Hospital, Sydney Local Health District, Concord, NSW 2139, Australia; Leonard.Kritharides@health.nsw.gov.au; 6 ANZAC Medical Research Institute, Faculty of Medicine, University of Sydney, Concord, NSW 2139, Australia; 7 Faculty of Medicine and Health, University of Sydney, Camperdown, NSW 2006, Australia; janette.vardy@sydney.edu.au; 8 Concord Cancer Centre, Concord Repatriation General Hospital, Concord, NSW 2139, Australia; 9 Calvary Mater Newcastle, Waratah, NSW 2298, Australia; Ina.Nordman@calvarymater.org.au (I.I.C.N.); Tony.Bonaventura@calvarymater.org.au (T.B.); 10 School of Medicine and Public Health, University of Newcastle, Callaghan, NSW 2308, Australia

**Keywords:** bevacizumab, VEGF inhibitors, cardiovascular risk, complications, outcomes

## Abstract

***Background:*** Bevacizumab, a vascular endothelial growth factor (VEGF) monoclonal antibody commonly used for the treatment of various cancers, is often associated with adverse cardiovascular effects such as hypertension, cardiac and cerebral ischemia, thrombosis, and bleeding events. Factors associated with increased risks of adverse cardiovascular effects with bevacizumab have not been intensively studied. In this study, we determined factors associated with hospital admissions due to cardiovascular complications in patients who received bevacizumab for cancer treatment. ***Methods and Results:*** We retrospectively collected data for all patients treated with bevacizumab between the 1st January 2016 and the 31st December 2017 at the Hunter New England Local Health District. Patients’ characteristics and their medical history were obtained from hospital electronic medical records. Outcome data were sourced from the Institutional Cardiac and Stroke Outcomes Unit database. A total of *n* = 230 patients (mean age 65, males *n* = 124 (53.9%)) were treated with bevacizumab during the study period. *N* = 28 patients were admitted to hospital for a major cardiovascular-related event. Higher total treatment dose (*p* < 0.05), concomitant hypertension (*p* = 0.005), diabetes (*p* = 0.04), atrial fibrillation (*p* = 0.03), and lack of use of statin therapy (*p* = 0.03) were key contributors to hospital admission. ***Conclusions:*** Results of our study highlight the fact that patients with concomitant baseline cardiovascular disease/risk factors are at an increased risk of cardiovascular hospitalization related to bevacizumab treatment. Careful baseline cardiovascular assessment may be an essential step to minimize cardiovascular complications.

## 1. Introduction

Cardiovascular disease (CVD) and cancer are by far the two most common disease conditions in the developed world and are therefore both major fields of concern in the health care system today. Generally, CVD and cancer are thought of as two separate entities. Development of highly effective cancer treatments has resulted in significant improvement in cancer outcomes. However, cancer survivors have an up to 15-fold higher risk of developing CVD [1], and it is now recognized as the leading cause of long-term morbidity and mortality among them. 

Bevacizumab, a recombinant vascular endothelial growth factor (VEGF) neutralizing antibody, is commonly used in the treatment of various cancers, including colorectal, cervical, ovarian, glioblastoma, and non-small cell lung cancers [2]. Bevacizumab binds to the VEGF-A ligand in the extracellular space preventing the ligand–receptor interaction and thus inhibiting the pro-angiogenic pathway that is often accelerated in tumor cell growth. Inhibition of VEGF-A by bevacizumab subsequently leads to deprivation of essential nutrients and oxygen to the tumor cells, promoting cell death [3]. It is the most widely used VEGF inhibitor and is used in the treatment of colorectal cancer, breast cancer, ovarian cancer, and many other advanced solid tumors [4].

Bevacizumab has been associated with cardiovascular complications such as hypertension, venous and arterial thromboembolism, left ventricular dysfunction, and less commonly, myocardial infarction and cerebrovascular events [5]. Overall, the incidence of any cardiotoxicity associated with bevacizumab treatment has been reported to be as high as 35% [6], with the predominant toxicity being hypertension. Mechanisms of bevacizumab-induced hypertension have been postulated to be due to reduced production of nitric oxide (NO) and increased endothelin-1, with VEGF inhibition production therefore leading to vasoconstriction [7]. These events also lead to increased peripheral vascular resistance resulting in elevated blood pressure. Inhibition of VEGF also causes a decrease in prostacyclin, which is involved in preventing platelet aggregation, and thus postulated to contribute to the development of arterial or venous thromboembolic events in bevacizumab-treated patients [7]. Hypertension, the commonest cardiovascular complication of bevacizumab therapy and a major cardiovascular risk factor, increases the risk of coronary and other vascular diseases as well as heart failure and arrhythmias, all of which generally increase risk of cardiovascular hospitalizations and adverse cardiovascular outcomes. 

There is a growing recognition of significant overlap between patients’ pre-existing cardiovascular risk factors (CVRF), cancer risk factors, and cancer therapy-associated cardiotoxicity [8]. All of these health issues increase the complexity of management of cancer patients and further highlight the lack of good quality data to guide best practice management and risk stratification of cancer patients from a cardiovascular point of view. While a number of studies have recorded various bevacizumab-associated toxicities [5], almost none have focused on predictors of cardiovascular admissions associated with those toxicities. Repeated cardiovascular-related hospitalizations are well associated with poor outcomes [9], and concerted efforts need to be made to understand and then mitigate the factors that lead to those in cancer patients. Better understanding of the impact of patients’ comorbidities on the development of major cardiovascular adverse events and hospitalization is critical to guide efforts to minimize these adverse outcomes.

Given these priorities and more widespread use of bevacizumab and other VEGF inhibitors, the major aim of this study is to evaluate the incidence and determinants of cardiovascular hospitalization following bevacizumab therapy.

## 2. Methods

### 2.1. Study Population

We retrospectively identified all patients who received anticancer therapy with bevacizumab in the Hunter New England Local Health District (HNELHD) from the 1st January 2016 through to the 31st December 2017 utilizing electronic medical and pharmacy records. The HNELHD region of New South Wales, Australia, covers an area of over 130,000 km^2^ and has a population of approximately 960,000, of whom approximately 45% live in metropolitan areas and 55% in regional or rural settings. The HNELHD has one major metropolitan teaching hospital, one Level 6 Cancer Hospital, a mix of several large regional centers, and many smaller regional centers. Cancer therapy is delivered at 5 of these centers, and patients from all these centers were included in the current study. Approximately 15% of the population were born overseas, and about 5% of the population are Aboriginal and Torres Strait Islanders [10]. There were no exclusion criteria. This study was approved by the Hunter New England Human Research Ethics Committee.

### 2.2. Data Collection

Demographic and administrative data as well as past medical history and co-morbidities from patients who received bevacizumab during the 2-year period were obtained from hospital electronic medical records. Total bevacizumab dose, cancer diagnosis, and concomitant chemotherapies were also recorded. 

Outcome data were sourced from the HNELHD Institutional Cardiac and Stroke Outcomes Unit database, which prospectively registers all public hospital admissions using consistent methodology (International Statistical Classification of the Diseases and Related Health Problems (ICD-10) codes). ICD-10 codes allow stratification as to the reason for admission and contributing diagnoses. In-hospital deaths and all-cause mortality are also recorded within the Hunter region. Patients’ presentations to emergency departments not resulting in admission were excluded. Comorbidities were identified from any ICD-10 code in the first 30 diagnoses on initial oncological evaluation and on discharge documentation of the hospital admission.

### 2.3. Statistical Analysis

Patient characteristics are reported as mean ± SD, median (interquartile range), or number (percentage). Categorical variables are presented as frequencies and percentages of total number of patients. Patients were divided into 2 groups: those with and those without unanticipated hospital admission due to cardiovascular complications. Between-group differences were compared using Student’s *t*-tests for means and χ^2^ analyses. A backward logistic regression analysis was performed to determine factors associated with cardiovascular hospital readmissions adjusted for age, body mass index (BMI), gender, total treatment dose, concomitant cardiovascular diseases, and medications. Goodness of fit in the final model was assessed using the Hosmer–Lemeshow test. Collinearity was assessed using Variance Inflation Factor for all parameters in the final model, and parameters with values below 5 were deemed to have no collinearity issues. All analyses were performed using the SPSS 25 System for Windows (IBM Corp, NY, Armonk, USA). Statistical significance was defined as a two-tailed *p*-value < 0.05.

## 3. Results

A total of 230 patients were prescribed bevacizumab between the 1st January 2016 and 31st December 2017. The commonest cancer diagnoses were colorectal (83%), ovarian (13%), and brain (4%) cancers. Patient baseline characteristics, co-morbidities, and previous pharmacological therapy are provided in Table 1. The study cohort had a mean age of 65 years, with 54% (*n* = 124) being male. More than half (57%) had a history of CVRF or CVD, including hypertension, atrial fibrillation, and dyslipidemia. ACE-inhibitors (ACEi) or angiotensin II receptor blockers (ARBs) were the most commonly used pharmacological agents reported in patients prior to bevacizumab treatment (*n* = 96, 42%). Sixty-eight patients (30%) had documented statin use, with 46 patients (20%) being on antiplatelet therapy. The mean total bevacizumab dose across the patient cohort was 7205 mg. 

A total of 28 patients were admitted to hospital with a cardiovascular diagnosis following bevacizumab treatment. Predominant primary admission diagnoses were atrial fibrillation (AF) and ischemic heart disease (IHD) (*n* = 14 and *n* = 8, respectively). There were 19 patients who were admitted within 6 months of starting bevacizumab, 6 patients within 6–18 months, and 3 patients after 24 months. Of these patients, 22 (79%) had an established history of hypertension, 8 (27%) had diabetes, and 5 (18%) suffered from atrial fibrillation. 

On univariate analyses, those with a history of cardiovascular disease, hypertension, diabetes, and atrial fibrillation, as well as those being treated with an ACEi/ARB, had an increased risk of CVD-related hospital admission (Table 2). There were no statistically significant differences between bevacizumab-treated patients with or without CVD-related hospital admission with respect to age, BMI, total treatment dose, dyslipidemia, IHD, cerebrovascular accident (CVA)/transient ischemic attack (TIA), prior CVD admission, or cancer history. Previous pharmacotherapy, including statin use, antiplatelets, beta-blockers, calcium channel blockers (CCB), and diuretics, also did not differ between patients with and without CVD-related hospital admission (Table 2). 

A backward stepwise logistic regression multivariable analysis to determine the key independent risk factors associated with CVD hospital admissions was performed (Table 3). Increased total bevacizumab dose (*p* = 0.048), concomitant hypertension (*p* = 0.005), diabetes (*p* = 0.039), and atrial fibrillation (*p* = 0.033) were key contributors to hospital admission. On the other hand, the odds ratio of CVD hospitalization for statin users over non-statin users was 0.28 (0.02–0.865; *p* = 0.027) after adjusting for other covariates. There was no evidence of collinearity in the final step of the model: the values of Variance Inflation Factor (VIF) for each of the final parameters in the model were all below 2.

## 4. Discussion

Advances in anticancer treatment have led to improved survival of patients with cancer but have been associated with an unintended increase in morbidity and mortality due to treatment side effects. Cardiovascular diseases are among the most frequent of these side effects, leading to premature morbidity and death among cancer survivors [11]. Newer and more targeted biological and immunological therapies have introduced new cardiotoxicity patterns due to their specific mechanisms of action and/or off-target effects [8]. VEGF inhibitors were amongst the earliest targeted cancer therapies to come onto the market, with bevacizumab being the key and the most commonly used member of this class of medications [12]. Although a more targeted treatment option, these medications are not selective to cancerous cells and their vasculature and therefore can have major impact on other vascular beds.

Among cancer survivors, bevacizumab, the most commonly used anticancer VEGF monoclonal antibody that inhibits angiogenesis, has been increasingly shown to be associated with risks of cardiovascular and cerebrovascular ischemia as well as bleeding events [5]. However, factors that predispose bevacizumab-treated patients to hospitalizations due to CVD have not been extensively investigated. 

In this study, we found that patients who received higher cumulative doses of bevacizumab as well as those with concomitant hypertension, diabetes, and atrial fibrillation were more likely to be hospitalized due to a CVD-related event. Furthermore, lack of use of cardioprotective statin therapy was also associated with increased risk of hospitalizations. The lack of appropriate guideline-directed use of statins has been previously reported for cancer survivors, despite the fact that these people have high CVD risk [13].

Bevacizumab has been extensively used since its first approval over a decade ago for the co-treatment of multiple cancers. Its major mechanism of action is inhibition of VEGF to reduce angiogenesis and thus promote cancer cell death due to lack of delivery of oxygen and nutrients to malignant cells [14]. However, VEGF inhibition is known to exert multiple actions that are detrimental to the cardiovascular system. Firstly, VEGF blockade results in endothelial dysfunction, leading to a decrease in nitric oxide formation in endothelial cells, subsequently leading to impaired vasorelaxation [15]. Secondly, inhibition of NO signaling can also contribute to increased platelet aggregation and adhesion [16], leading to exaggerated or inappropriate thrombosis. Taken together, these actions result in the development of hypertension, cardiovascular and cerebrovascular ischemia, and increased risk of bleeding due to endothelial defects that expose procoagulant phospholipids on the luminal plasma membrane or underlying matrix [15]. 

Accumulating clinical and preclinical data suggest an interdependence and biological overlap based on commonality of risk factors (smoking, obesity, aging, and diet) and pathophysiological mechanisms (inflammation, redox stress, abnormal metabolism, and mitochondrial dysfunction) between cancer and cardiovascular disease. 

Many studies have reported the incidence of various bevacizumab-related adverse cardiovascular events [4]. Hypertension has been the most widely reported adverse effect with incidence ranging from 3% [17] to 31.8% [18], depending on the study type, participants’ characteristics, and dose of bevacizumab given. Vascular thromboembolic events are the next commonest category of adverse events reported, which may translate into the observed higher incidence of coronary and peripheral arterial events seen in bevacizumab-treated patients [5]. The incidence of bevacizumab-associated venous thrombolytic events varied from 2% [19] to 11.9% [20]. However, a larger study with over 6000 participants concluded that upon adjustments for patient risk factors and tumor types, the increase in venous thrombolytic events was not directly related to bevacizumab use [21]. The incidence of arterial thrombolytic events associated with bevacizumab use ranges in the literature from 2.4% [22] to 6.8% [18]. However, similarly to venous thrombolytic events, it has also been suggested that this is more dependent on age and previous history of thromboembolic events [23], rather than bevacizumab use alone. The incidence of heart failure in the setting of bevacizumab use has been less frequently reported, but seems to be around 4%, and as high as 14% when bevacizumab was used in combination therapy [17]. One study, however, reported a very high incidence of 35% when bevacizumab was combined with doxorubicin-containing chemotherapy [6]. Despite the above statistics, there is a marked paucity of data on the predictors (rather than incidence) of cardiotoxicity associated with bevacizumab, and in particular predictors of cardiovascular hospitalizations as a surrogate for significant cardiotoxicity. 

In the current study, we observed a 12% rate of hospital admission associated with major cardiovascular events within a two-year period in patients treated with bevacizumab. Most of these patients had concomitant CVD prior to initiation of bevacizumab. A previous meta-analysis showed a higher number of bevacizumab cycles, corresponding to a higher total dose, to be associated with increased risks of cardiovascular events [24]. In the current study, we also observed that higher doses of bevacizumab were associated with increased risks of hospitalization due to CVD. It is possible that differences in cancer types and stages of cancer, with other concomitant treatment types, are potential confounders of this finding. Given the wide-ranging indications for bevacizumab use for different cancers, it would be difficult to adjust for these confounders. 

Our study has several limitations: the cohort was relatively small and relied on the accuracy of cardiovascular adverse event coding. However, the cohort had diverse socioeconomic and geographic representation due to the nature and location of the facilities within our health district. As only major CVD complications would have led to hospital admission, the true rate of CVD complications is likely to be underestimated; for example, development of accelerated hypertension as a consequence of bevacizumab therapy would not require hospitalization and thus would not be captured in our dataset. The size of the cohort and predominance of colorectal cancer etiology (>80%) did not allow us to take into account different types of cancer in the analyses. We only recorded the first hospital admission due to CVD and did not take into account multiple hospital admissions. Finally, it is possible that the CVD readmissions were not entirely due to bevacizumab alone as we could not account for other concomitant cancer treatments. 

The integrative approach of cardio-oncology is increasing in demand to address a rising incidence/prevalence of CVD in a growing number of cancer patients and survivors. Currently, baseline cardiovascular risk assessment and optimal control of CVD risk with appropriate preventative medications are not routinely and consistently performed in cancer patients upon initiation of their cancer treatments, and approaches vary from center to center. 

Developing strategies to predict and reduce the risk of future events with anticancer therapies, especially with VEGF inhibitors, is clearly needed. The results of our study highlight that patients with concomitant baseline CVD are at increased risk of hospitalization due to CV adverse events after cancer treatment. In fact, the strongest determinants of subsequent cardiovascular admission are pre-existing CVRF, while total bevacizumab dose is of marginal statistical significance, and prior statin therapy is protective. Thus, more intensive follow-up of those patients with greater baseline risk for CV events, a low threshold for prescription of statins if compatible with guideline-based recommendations, and reviewing the relative benefits and risks of higher total doses of bevacizumab for individual patients may need to be considered. As the univariate association of ACE inhibitor/ARB usage with CV events was no longer apparent after multivariable adjustment, this is most likely a reflection of treated hypertension or prior congestive heart failure rather than a true adverse effect of these agents.

While investigations into the potential benefits of primary preventative cardioprotective therapies for individual chemotherapeutic regimes are required, careful baseline CVD assessment of patients is an essential step to prevent unexpected CV complications that may result in interruption of a patient’s cancer treatments or development of CVD upon cancer survival. This message is reiterated in the just-published position statement from the Cardio-Oncology Study Group of the Heart Failure Association of the European Society of Cardiology with the International Cardio-Oncology Society [25]. Our study further highlights the need for consistent recording and management of CVD and CV factors in cancer patients at every stage of the cancer trajectory, from diagnosis into survivorship.

## Figures and Tables

**Table 1:** Baseline patient characteristics.

Grouped Characteristics	Variables
No of Patients, *n*	230
Deceased Patients (as of 01/11/2019), *n* (%)	148 (64)
Male Gender, *n* (%)	124 (54)
Age (years), mean ± SD	65 ± 13
BMI (kg/m^2^), mean ± SD	26 ± 6
Smoking (current/past, *n* (%)	148 (64)
Total Bevacizumab Dose (mg), mean ± SD	7205 ± 6657
Number of Bevacizumab cycles, mean ± SD	14 ± 12
**Co-Morbidities**	
Hypertension, *n* (%)	122 (53)
Dyslipidemia, *n* (%)	64 (28)
Diabetes mellitus, *n* (%)	35 (15)
IHD, *n* (%)	19 (8)
Atrial Fibrillation, *n* (%)	15 (6)
CVA/TIA, *n* (%)	16 (7)
CVD History, *n* (%)	132 (57)
Prior CVD Admission, *n* (%)	34 (15)
Previous Cancer, *n* (%)	19 (8)
**Pharmacological Therapy**	
Statins, *n* (%)	68 (30)
Antiplatelets, *n* (%)	46 (20)
Beta-blockers, *n* (%)	40 (17)
ACE Inhibitors/ARBs, *n* (%)	96 (42)
Calcium Channel Blockers, *n* (%)	40 (17)
Diuretics, *n* (%)	32 (14)

SD—standard deviation, BMI—body mass index, IHD—ischemic heart disease, CVA—cerebrovascular accident, TIA—transient ischemic attack, CVD—cardiovascular disease, ACE—angiotensin-converting enzyme, ARBs—angiotensin II receptor blockers.

**Table 2:** Univariate analysis results.

	No CVD Admission (*n* = 202)	CVD Admission (*n* = 28)	*p*-Value
Female Gender, *n* (%)	97 (48)	9 (32)	0.114
Male Gender, *n* (%)	105 (52)	19 (68)	0.114
Age (years), mean ± SD	64.76 ± 13.09	68.5 ± 10.24	0.094
BMI (kg/m^2^), mean ± SD	26.05 ± 5.97	27.29 ± 6.23	0.34
Total Bevacizumab Dose (mg), mean ± SD	6801.24 ± 6500.53	9061.61 ± 7241.74	0.130
Chemo Commencement Till Death (days), mean ± SD	430.58 ± 10.42	461 ± 294.88	0.712
Deceased Patients, *n* (%)	130 (64.36)	18 (64.29)	1
Hypertension, *n* (%)	100 (50)	22 (79)	0.004
Dyslipidemia, *n* (%)	55 (27)	9 (32)	0.653
Diabetes mellitus, *n* (%)	27 (13)	8 (29)	0.048
IHD, *n* (%)	15 (7)	4 (14)	0.262
Atrial Fibrillation, *n* (%)	10 (5)	5 (18)	0.024
CVA/TIA, *n* (%)	14 (7)	2 (7)	1
CVD History, *n* (%)	110 (54)	22 (79)	0.023
Prior CVD Admission, *n* (%)	28 (14)	6 (21)	0.269
Previous Cancer, *n* (%)	17 (8)	2 (7)	1
Statins, *n* (%)	61 (30.2)	7 (25)	0.663
Antiplatelets, *n* (%)	38 (19)	8 (29)	0.218
Beta-blockers, *n* (%)	32 (16)	8 (29)	0.111
ACE Inhibitors/ARBs, *n* (%)	79 (39)	17 (61)	0.040
CCB, *n* (%)	32 (16)	8 (29)	0.111
Diuretics, *n* (%)	25 (12)	7 (25)	0.082

SD—standard deviation, BMI—body mass index, IHD—ischemic heart disease, CVA—cerebrovascular accident, TIA—transient ischemic attack, CVD—cardiovascular disease, ACE—angiotensin-converting enzyme, ARBs—angiotensin II receptor blockers, CCB—calcium channel blockers.

**Table 3:** Multivariable analysis results.

Parameter	β	*p*-Value	Odds Ratio [Exp (B)]	95% CI
Total Bevacizumab Dose (mg)	0.001	0.048	1.001	1.001–1.001
Hypertension	1.467	0.005	4.336	1.54–12.21
Diabetes	1.154	0.039	3.170	1.06–9.479
Atrial Fibrillation	1.359	0.033	3.894	1.117–13.576
Statin use	−1.268	0.027	0.281	0.092–0.865
